# Properties of resistant cells generated from lung cancer cell lines treated with EGFR inhibitors

**DOI:** 10.1186/1471-2407-12-95

**Published:** 2012-03-20

**Authors:** Gargi Ghosh, Xiaojun Lian, Stephen J Kron, Sean P Palecek

**Affiliations:** 1 Department of Chemical and Biological Engineering, University of Wisconsin, Madison, 1415 Engineering Drive, Madison, WI 53706, USA; 2 Ludwig Center for Metastasis Research, University of Chicago, Chicago, IL 60637, USA; 3 Department of Mechanical Engineering, University of Michigan, Dearborn, MI 48128, USA

**Keywords:** EGFR tyrosine kinase, Erlotinib, Cancer stem cells, Tumor spheroids, Side population

## Abstract

**Background:**

Epidermal growth factor receptor (EGFR) signaling plays an important role in non-small cell lung cancer (NSCLC) and therapeutics targeted against EGFR have been effective in treating a subset of patients bearing somatic EFGR mutations. However, the cancer eventually progresses during treatment with EGFR inhibitors, even in the patients who respond to these drugs initially. Recent studies have identified that the acquisition of resistance in approximately 50% of cases is due to generation of a secondary mutation (T790M) in the EGFR kinase domain. In about 20% of the cases, resistance is associated with the amplification of MET kinase. In the remaining 30-40% of the cases, the mechanism underpinning the therapeutic resistance is unknown.

**Methods:**

An erlotinib resistant subline (H1650-ER1) was generated upon continuous exposure of NSCLC cell line NCI-H1650 to erlotinib. Cancer stem cell like traits including expression of stem cell markers, enhanced ability to self-renew and differentiate, and increased tumorigenicity *in vitro *were assessed in erlotinib resistant H1650-ER1 cells.

**Results:**

The erlotinib resistant subline contained a population of cells with properties similar to cancer stem cells. These cells were found to be less sensitive towards erlotinib treatment as measured by cell proliferation and generation of tumor spheres in the presence of erlotinib.

**Conclusions:**

Our findings suggest that in cases of NSCLC accompanied by mutant EGFR, treatment targeting inhibition of EGFR kinase activity in differentiated cancer cells may generate a population of cancer cells with stem cell properties.

## Background

Recent years have seen the emergence of therapeutics directed against specific signaling pathways critical for the onset and progression of cancer. Protein tyrosine kinases (PTKs), by the virtue of their regulation of cellular functions that contribute to cancer, including cell proliferation, survival, apoptosis, migration, and DNA damage repair, have emerged as new anticancer targets. Rational targeting of PTK activity to control these signaling pathways, and thus correct aberrant cellular behaviors in cancer, has been successful in improving outcomes of many types of cancer [1]. Moreover, the specificity of these targeted drugs results in fewer and less severe side effects compared to conventional cancer treatments which are non specific in their actions. Of the approximately 20 classes of PTKs, the epidermal growth factor receptor (EGFR) family, whose members include HER1 (EGFR), HER2/neu (ErbB2), HER3 (ErbB3), and Her4 (ErbB4) [2], has been the most widely studied. While the EGFR signaling cascade is essential for homeostasis, dysregulation of EGFR kinase activity has been implicated in the oncogenic transformation of cells [3,4]. EGFR overexpression, gene amplification, mutations, and increased kinase activity have been observed in many solid cancers of epithelial origin including breast, lung, head and neck, ovarian, bladder, and pancreatic cancers [2,5].

Specifically, frequent abnormal amplification or activation of EGFR has been observed in non-small cell lung cancer (NSCLC). Two small molecule EGFR tyrosine kinase inhibitors (EGFR-TKI), gefitinib (Iressa, AstraZeneca International) and erlotinib (Tarveca, OSI Pharmaceuticals) have been evaluated in patients with NSCLC [6,7]. These ATP competitive, reversible EGFR-TKIs have been effective only in a small subset of NSCLC patients bearing somatic mutations (deletions in exon 19 and the L858R mutation) in the kinase domain of EGFR [8]. Nevertheless, patients initially responding to TKI therapy invariably develop resistance to these drugs, thereby limiting progression-free survival to approximately 9-13 months with a median survival of 2 years [9]. In the past several years, studies underpinned the molecular mechanisms responsible for drug resistance including acquisition of secondary mutation in EGFR kinase domain (threonine to methionine mutation, T790M) and/or c-MET amplification [10-13]. However, these constitute only ~50-70% of EGFR-TKI resistant cases, indicating mechanisms leading to resistance in the remaining cases are yet to be unraveled. Recent endeavors have identified that in addition to increased receptor internalization or altered EGFR trafficking [14], epithelial to mesenchymal transition (EMT) can be related with acquisition of resistance towards EGFR TKIs [15-18].

EMT, characterized by the loss of cell-cell junctions, repression of E-cadherin expression and gain of mesenchymal markers significantly contributes to cancer invasion and metastasis. Recent evidence indicates EMT induction in tumor cells can also lead to emergence and/or enrichment of cancer stem cells (CSCs) [19]. CSCs, also known as tumor initiating cells or cancer stem like cells, refer to a minor subpopulation of cancer cells with properties similar to somatic stem cells including self-renewal and multi-lineage differentiation. Initially identified in acute myeloid leukemia, CSCs have later been found in various cancers including breast, lung, brain, pancreatic, and prostate cancer [20-27]. By the virtue of altered cell cycle kinetics, increased DNA repair response, increased expression of antiapoptotic regulators as well as transporter proteins, CSCs are able to survive radiation or chemotherapeutic insults [28]. Thus, these cells are more refractory to cytotoxic agents compared to the differentiated cancer cells which constitute the bulk of the tumor. In fact it is believed that CSCs contribute significantly to tumor relapse following chemo or radiotherapy.

Based on these observations, we speculated that CSC selection during prolonged exposure to EGFR TKIs may play a role in eventual progression of cancer after a period of successful response. Recent evidence shows existence of a population of cells expressing cancer stem cell markers CD44^high^/CD24^low ^in erlotinib resistant non small cell lung cancer (NSCLC) cell lines [15]. However, to the best our knowledge these cells were not characterized in terms of their potential to self-renew, differentiate or induce resistance to EGFR-TKI therapy. In this study we generated an erlotinib resistant subline (H1650-ER1) from erlotinib sensitive lung cancer cell line NCI-H1650. Enrichment of cells with CSC markers and phenotypes in the resistant subline was confirmed by several techniques: (a) expression profiling of cell surface markers, (b) side population (SP) analysis (identification of a population of cells, called SP, characterized by high efflux of DNA-binding dye, Hoechst 33342 or DyeCycle Violet (DCV) dye by ABCG2, an ATP binding cassette transporter [29,30]) and (c) culture of cells in suspension in serum free medium to promote generation of tumor spheroids.

Our studies demonstrate that the erlotinib resistant subline was composed of an increased population of cancer stem cell-like cells and exhibited enhanced colony formation ability in soft agar. SP cells isolated from H1650-ER1 showed self-renewal as well as differentiation potential. Furthermore, SP cells were more resistant to EGFR-TKIs than non-SP cells. These observations indicate that resistance to molecular targeted therapy could arise from selection and enrichment of cancer stem cell-like cells, which are intrinsically resistant to erlotinib.

## Methods

### Cells

Human lung cancer cell line NCI-H1650 (hence forth referred to as H1650) was obtained from ATCC (Manassas, VA). The cells were maintained in RPMI-1640 supplemented with 10% FBS and glutamine. During culture, the medium was changed every other day. The cells were passaged every 5-6 days using Trypsin-EDTA (0.25% trypsin, 1 mM EDTA). Generation of the H1650-ER1 subline has been described previously [31]. Briefly, starting with an erlotinib (LC labs, Woburn, MA) concentration of 2.5 μM, the exposure dose was doubled every 15 days until a final concentration of 20 μM was achieved. The cells were maintained in continuous culture at of 20 μM erlotinib for 30 days. Then the resistance phenotype of the pools was characterized by a cell proliferation assay. The resistant pool was then used to establish individual clones. The established clones were further maintained in culture with 20 μM erlotinib for another 30 days. Cell viability was then measured following exposure to varying concentrations of erlotinib. Prior to any experiment, the cells were cultured in medium lacking erlotinib for at least a week.

Human head and neck squamous cell carcinoma cell line SCC-1 and erlotinib and gefitinib resistant sublines (SCC-1-Erl-R and SCC-1-Gef-R) were maintained in DMEM supplemented with 10% FBS, and 1 μg/mL hydrocortisone.

### Cell Migration Assay

H1650 and H1650-ER1 cells were seeded in each well of 6 well plates and allowed to reach confluence. Once confluent, a wound was inflicted in the monolayer by scraping with a sterile 200 μL pipette tip. The cell monolayer was then washed three times with DPBS to remove the cell debris and incubated with the growth media. Pictures of the wound were captured at time points t = 0 and t = 12 h to calculate the wound area. Migration of the cells was calculated fractional closure of the wound area.

### Spheroid formation assay

Liquid overlay culture was used to investigate the capacity of the cells to form spheroids. For the purpose, each well of 6 well plates was covered with a thin film of 1% agarose in serum free DMEM/F12 medium. Cell monolayers were dissociated with trypsin-EDTA into single cells and resuspended in DMEM/F12 (Invitrogen, Carlsbad, CA) medium supplemented with human recombinant epidermal growth factor (EGF; 10 ng/ml) and basic fibroblast growth factor receptor (bFGF; 10 ng/ml) and plated in agarose coated 6 well plates. The medium was replaced every 3 days. In order to assess self renewal through formation of secondary spheroids, the spheroids were collected by centrifugation, dissociated into single cells by treating with trypsin and passing through 40 μm cell strainer, and then cultured under conditions described above.

### SP analysis

To identify SP cells, cells were stained with DyeCycle Violet (DCV) stain (Invitrogen, Carlsbad, CA) using methods modified from Telford et al [30]. Briefly, cells at a density of 10^6 ^cells/ml were incubated with DCV dye (10 μM) with or without 50 μM verapamil (Sigma, St. Louis, MO) at 37°C for 90 min with intermittent shaking. At the end of the staining, the cells were washed in ice cold PBS and resuspended in ice cold RPMI-1640 medium. Propidium iodide at the final concentration of 2 μg/ml was added to the cells to gate viable cells and the cells were immediately placed in ice. Analysis was carried out on a BD LSR II flow cytometer or flow sorted on a BD FACSAria (BD Biosciences, San Jose, CA). DCV dye was excited by violet diode laser (408 nm) and its fluorescence was dual wavelength analyzed (blue 450/40 nm; red 650 nm LP).

In order to investigate the ability of SP cells to differentiate, sorted SP and non SP cells were cultured in RPMI 1640 for 10 days. The cells were then stained with DCV dye and the SP fraction of the two subpopulations was determined.

### Soft-agar assay

To determine the anchorage independent growth potential, colony formation in soft agar was measured. For the base layer, 1 mL of 0.5% of agar in RPMI 1640 was added in each well of 6 well plates. A top layer consisting of 2500 cells suspended in 0.35% agar in RPMI 1640 was plated on top of the base layer. Agar plates were incubated at 37°C for 2 weeks. Growth medium (RPMI1640 supplemented with 10% FBS and 2 mM glutamine) was changed every 3 days. After 2 weeks, the colonies were stained with 0.005% crystal violet and colonies > 20 μm were counted. Three independent assays were performed in duplicate.

### Cell viability assay

SP and non SP cells sorted from H1650 and H1650-ER1 cells were seeded at a density of 5 × 10^3 ^cells/well in 96 well plates. After 24 hr, erlotinib at varying concentrations was added and the cells were incubated further for 48 hr. The cells were then washed with PBS and cell viability was measured using a XTT assay kit (Sigma, St. Louis, MO).

### Quantitative RT-PCR

Quantitative RT-PCR (qRT-PCR) was conducted to examine the mRNA expression of E-cadherin, vimentin, occludin, fibronectin, OCT3/4, NANOG, SOX-2, ID2 and GAPDH in H1650 and H1650-ER1 cells. The mRNA expression of OCT3/4, NANOG, BMI1 and STAT3 was investigated in H1650-ER1 cells, H1650-ER1 spheroids and adherent cells. Total RNA from the cells were extracted using RNeasy Mini kit (Qiagen, Valencia, CA) and cDNA was generated using high capacity cDNA reverse transcription kit (Applied Biosystems). qRT-PCR was performed with SYBR Green PCR master mix (Applied Biosystems, Carlsbad, CA) following manufacturer's instructions. Gene expression in H1650, H1650-ER1 cells, H1650-ER1 spheroids and adherent cells was initially normalized against GAPDH to obtain ΔC_t _values. Relative fold change in gene expression was then compared between H1650-ER1 and H1650 or H1650-ER1 spheroids, adherent cells and H1650-ER1 cells using ΔC_t _method of quantitation. ΔC_t _values of different cell populations were used to performstatistical analysis. p-value < 0.05 was considered significantly different. The primers are listed in Table 1.

**Table 1:** Sequences of oligonucleotide primers used in this study

Gene	Forward	Reverse
E-cadherin	5'-AGGAATTCTTGCTTTGCTAATTCTG	5'-CGAAGAAACAGCAAGAGCAGC

Vimentin	5'-GAGAACTTTGCCGTTGAAGC	5'-CTAACGGTGGATGTCCTTCG

Fibronectin	5'-GTT GTT ACC GTG GGC AAC TC	5'-CTG ACG GTC CCA CTT CTC TC

Occludin	5'-TTGGGACAGAGGCTATGG	5'-ACCCACTCTTCAACATTGGG

Snail	5'-TTCCAGCAGCCCAACGACCAG	5'-CGGACTCTTGGTGCTTGTGGA

Twist	5'-GGAGTCCGCAGTCTTACGAG	5'-TCTGGAGGACCTGGTAGAGG

Oct3/4	5'-CAGTGCCCGAAACCCACAC	5'-GGAGACCCAGCAGCCTCAAA

SOX2	5'-CAAGATGCACAACTCGGAGA	5'-GTTCATGTGCGCGTAACTGT

NANOG	5'-CAGAAGGCCTCAGCACCTAC	5'-ATTGTTCCAGGTCTGGTTGC

ID2	5'-GACCCGATGAGCCTGCTATAC	5'-AATAGTGGGATGCGAGTCCAG

BMI-1	5'-GATGCCACAACCATAATAGAA	5'-TCATTCACCTCCTCCTTAGAT

STAT3	5'-GGGTGGAGAAGGACATCAGCGGTAA	5'-GCCGACAATACTTTCCGAATGC

### Immunofluorescence

H1650 and H1650-ER1 cells were fixed in 4% paraformaldehyde for 15 min at 37°C before blocking and permeabilizing with 5% milk in phosphate-buffered saline (PBS) containing 0.4% Triton X-100. Then the cells were incubated overnight with anti-β-catenin antibody (Cell Signaling Technology, Danvers, MA) at 4°C. Next, the cells were stained with the Alexa 488 fluorophore-conjugated secondary antibody (Invitrogen, Carlsbad, CA) and DAPI for 1 hr at room temperature. Immunofluorescence images were examined with an epifluorescence microscope (Leica DM IRB) and imaged using QImaging Retiga 4000R camera.

### Flow analysis

H1650 and H1650-ER1 cells (2 × 10^5^) were fixed in 1% paraformaldehyde for 10 min at 37°C and then incubated overnight with Alexa647-CD24 (BD Biosciences), FITC-CD44 (BD Biosciences), APC-CD133 (Miltenyi Biotec), PE-anti-SSEA-3 (BD Pharmingen), SSEA-4 (Santa Cruz), Tra-1-60 (Santa Cruz), and Tra-1-80 antibodies (Santa Cruz) (1:500 in PBS with 2% FBS and 0.1% NaN_3_) at 4°C. After 30 min of secondary stain with Alexa 488 anti-mouse IgG secondary antibody (for SSEA-4 stained cells), and PE-anti-mouse IgM antibody (for Tra-160 and Tra-1-80), cells were analyzed on BD LSR II flow cytometer. Control samples were incubated with only secondary antibody or APC-mouse IgG and PE-rat IgM antibodies.

## Results and discussion

### Characterization of an erlotinib resistant cell line

**Figure 1:**
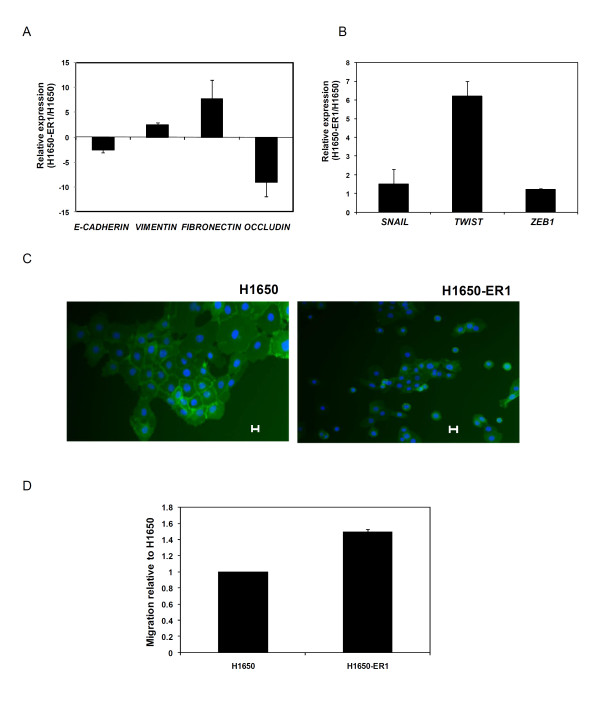
**Characterization of H1650-ER1 cells**. (A) mRNA expression of E-cadherin, vimentin, occludin and fibronectin in H1650 and H1650-ER1 cells was measured by quantitative RT-PCR. Fold change expression was normalized with respect to H1650 cells. (B) mRNA expression of Snail, Twist and Zeb1 in H1650 and H1650-ER1 cells was measured by quantitative RT-PCR. Fold change expression was normalized with respect to H1650 cells. Error bars represent s.e.m. (n = 3). (C) Immunofluorescence of H1650 and H1650-ER1 cells stained with DAPI (blue) and antiβ-catenin antibody (green). (D) H1650 and H1650-ER1 cells were seeded on 6 well plates. After 48 hr, a scratch was induced in the confluent cell monolayer. Images were obtained at time t = 0 and after 12 hr (t = 12) to monitor cell migration. Percent cell migration was calculated based on migration of H1650 cells. The error bars represent s.e.m. (n = 3).

An erlotinib resistant NCI-H1650 subline (H1650-ER1) was generated by progressively exposing the cells to increasing concentrations of erlotinib [31]. The resistant phenotype was characterized by quantifying cell viability at different concentrations of erlotinib and also via a clonogenic assay. Sequencing of the EGFR gene revealed the persistence of the deletion mutation ΔE746-A750 within the EGFR kinase domain in both H1650 and the resistant H1650-ER1 subline; however, no additional mutation was observed in the EGFR open reading frame in H1650-ER1 cells. Moreover, MET amplification, often associated with acquired erlotinib or gefitinib resistance, was not observed. Since cells with a mesenchymal phenotype are generally more resistant to EGFR-TKI treatment than cells with an epithelial phenotype, as shown in both *in vitro *studies and clinical samples [16,32,33], we analyzed the gene expression profile of epithelial and mesenchymal markers in H1650 and H1650-ER1 cells. There was a striking difference in the expression of genes associated with an epithelial to mesenchymal transition (EMT). While expression of E-cadherin and occludin were downregulated, expression of vimentin and fibronectin were upregulated in H1650-ER1 cells compared to parental cell line (p-value < 0.005) (Figure 1A). Moreover, the transcription factors Snail, Twist, and Zeb, which are known to promote transition of cells toward a mesenchymal phenotype, were also upregulated in H1650-ER1 cells as compared to H1650 cells (p-value < 0.05) (Figure 1B). Immunofluorescence analysis showed that β-catenin remained localized at the membranes in 68% of H1650 cells as opposed to 33% of H1650-ER1 cells (p-value < 0.01), whereas there was greater cytoplasmic localization of β-catenin in H1650-ER1 cells (51% of H1650-ER1 cells vs. 18% of H1650 cells, p-value < 0.01) (Figure 1C). In addition, resistant cells also displayed enhanced motility (p-value < 0.05) measured as the ability to heal a defect in a cell monolayer (Figure 1D). However, there was no obvious change in morphological phenotype between H1650 and H1650-ER1 cells. Taken together our observations suggest that H1650-ER1 cells have undergone a partial EMT.

### Analysis of CSC and embryonic stem cell markers

**Figure 2:**
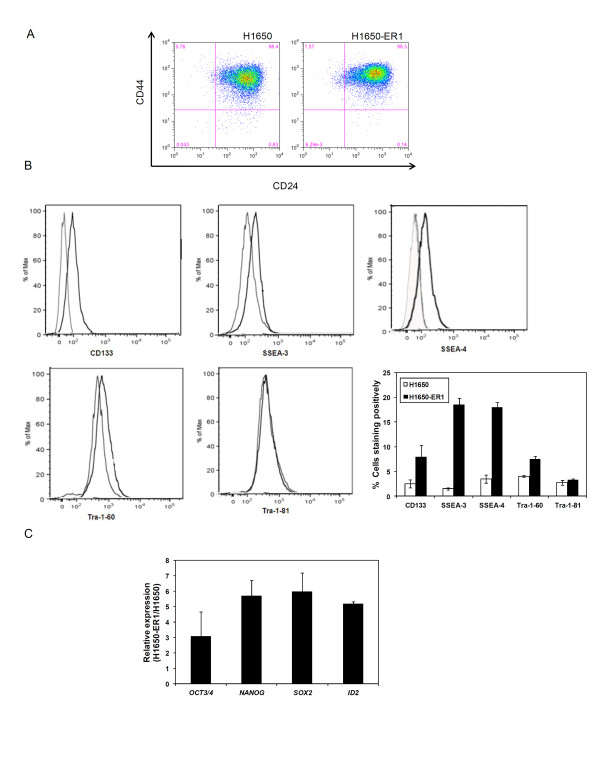
**Expression of stem cell markers**. (A) Analysis of CD44 and CD24 expression in H1650 and H1650-ER1 cells by flow cytometry. (B) Flow cytometric analysis of CD133, SSEA-3, SSEA-4, Tra-1-60, and Tra-1-81 expression in H1650 and H1650-ER1 cells. Quantification of cells staining positively for different markers. The error bars represent s.e.m. (n = 3). H1650-ER1 cells were enriched for CD133+, SSEA-3+, SSEA-4+, and Tra-1-60+ populations as compared to parental H1650 cells (p-value < 0.05). No differential expression between H1650 and H1650-ER1 cells was observed for Tra-1-81. (C) mRNA expression of OCT3/4, NANOG, SOX-2, and ID2 in H1650 and H1650-ER1 cells was measured by quantitative RT-PCR. Fold change expression was normalized with respect to H1650 cells. The error bars represent s.e.m. (n = 3).

To characterize whether H1650-ER1 cells are enriched with a cell population possessing stem cell properties, we analyzed the expression of CSC surface markers CD24, CD44, and CD133 and embryonic stem cell markers including SSEA-3, SSEA-4, Tra-1-60 and Tra-1-81. As demonstrated in Figure 2A, approximately twice as many H1650-ER1 cells displayed CD44^high^/CD24^low ^expression patterns as compared to H1650 cells. The CD44^high^/CD24^low ^cells comprise a small fraction of the total H1650-ER1 population, representing less than 2% of the cells. Expression of CD133 has been found in stem cells of several cancers including lung, brain, prostate and pancreatic cancer [22,23,34-37]. Our study revealed that resistant H1650-ER1 cells were substantially enriched for CD133+, SSEA-3+, SSEA-4+, and Tra-1-60+ populations as compared to parental H1650 cells (Figure 2B). No differential expression between H1650 and H1650-ER1 cells was observed for Tra-1-81.

mRNA expression levels of transcription factors OCT3/4, NANOG, SOX-2, and inhibitor of differentiation 2 (ID2) were compared among H1650 and H1650-ER1 cells. These genes encode proteins involved in self-renewal of undifferentiated stem cells and all of these genes were expressed to a greater extent in H1650-ER1 cells (p-value < 0.05) than in the parental H1650 cells (Figure 2B). While expression of individual stem cell markers, including OCT4, in somatic and cancer stem cells has been questioned [38], a role of OCT4 and NANOG expression in regulating epithelial-mesenchymal transitions, tumor-initiating ability, and metastasis in lung adenocarcinomas has been reported [39]. Taken together, these experiments indicate that the H1650-ER1 resistant subline expresses markers that have been associated with various adult and pluripotent stem cells at a higher level than the parental H1650 cell line. However, the roles of these genes in mediating H1650-ER1 phenotypes remain unclear.

### Detection of tumor spheroid cells with self-renewal capability

**Figure 3:**
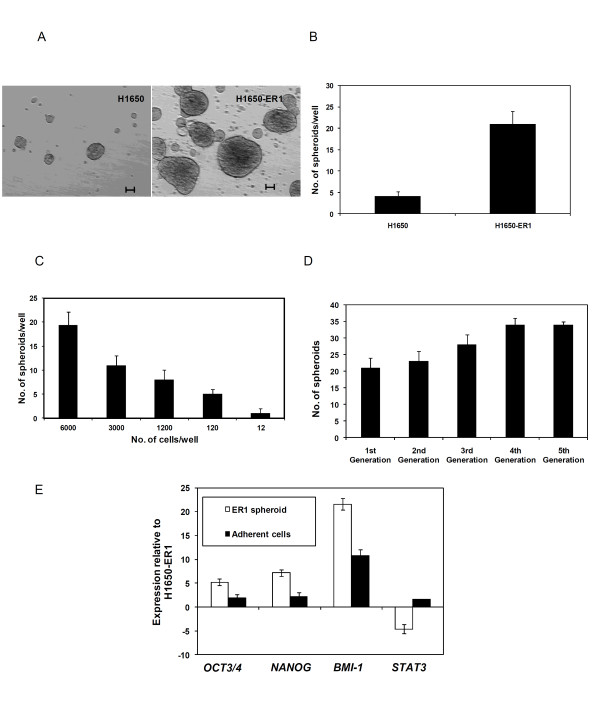
**H1650 and H1650-ER1 cells were cultured under non-adherent conditions in serum free medium and spheroids were counted after 15 days**. (A) Images of spheroids generated by H1650 and H1650-ER1 cells. The scale bar corresponds to 50 μm. (B) Quantification of spheroids generated. H1650 and H1650-ER1 cells were seeded in 6 well plates at a density of 6000 cells per well. The resistant subline generated a significantly higher number of spheroids (p-value < 0.01). Each data point represents the mean of three independent experiments. The error bars represent s.e.m. (n = 3). (C) Clonogenicity of H1650-ER1 cells determined by limiting dilution assay. Cells were seeded in 6 well plates at density varying from 12-6000 cells per well. Each data point represents the mean of 6 wells. The error bars represent S.D. (n = 6). (D) Self renewal ability was determined by dissociating spheres into single cells, replating them under non adherent conditions, and counting generation of secondary spheres after 15 days. Each data point represents the mean of 6 replicates. The error bars represent S.D. (E) mRNA expression of OCT3/4, NANOG, BMI-1 and STAT3 in H1650-ER1, ER1 spheroids (3^rd ^generation) and adherent cells were measured by quantitative RT-PCR. Fold change expression was normalized with respect to H1650-ER1 cells. The error bars represent s.e.m. (n = 3).

One critical property of stem cells is their ability to self-renew. We evaluated the self renewal properties of H1650-ER1 cells by the ability of individualized cells to form spheroids when seeded in agarose and cultured in serum free medium supplemented with EGF and bFGF. Within 48 h, cells formed three dimensional aggregates and eventually generated spheroids (Figure 3A). Some cellular aggregates of H1650 cells were observed after 48 h, but the vast majority of these aggregates collapsed and disintegrated within a few days. Few of these aggregates gave rise to spheroids (Figure 3A). As shown in Figure 3B, the spheroid formation frequency of H1650-ER1 (21 ± 3 spheroids/6 × 10^3 ^cells) was significantly higher than that of H1650 (4 ± 1 spheroids/6 × 10^3 ^cells) cells at day 15 (p-value < 0.01). Moreover, as observed in Figure 3A, much larger spheroids were formed by H1650-ER1 cells (170 ± 22 μm) than the parental cells (78 ± 6 μm). The clonogenicity of H1650-ER1 cells was demonstrated by limiting dilution experiments which revealed that ~1 in 12 cells possessed the capacity to give rise to a spheroid (Figure 3C).

To further investigate the self renewal potential, spheroids from H1650-ER1 cells were dissociated into single cells and cultured under non adherent conditions for 5 generations. Spheroids from all generations formed secondary spheroids. As shown in Figure 3D, compared to the first generation, significantly more H1650-ER1 spheroids were observed after serially passaging in culture, confirming the self-renewal ability of the resistant subline. (p-value < 0.05 for 3^rd^, 4^th ^and 5^th ^generations).

Dissociated single cells from spheroids were also cultured in adherent conditions in RPMI 1640 containing 10% FBS to induce differentiation. Expression of the genes OCT3/4, NANOG, BMI-1 and STAT3, which are associated with pluripotency in stem cells [40-42], was evaluated by RT-PCR in H1650-ER1 cells, H1650-ER1 tumor spheroids, and adherent cells. As expected, compared to H1650-ER1 cells, spheroids showed greater expression of OCT3/4, NANOG and BMI-1 (p-value < 0.05), suggesting a more stem like character for this subgroup (Figure 3E). However, STAT3 expression was downregulated. The reduced expression levels of these genes in adherent cells suggest that the cells have started to differentiate; levels of OCT3/4 and NANOG in adherent cells were not significantly different from H1650-ER1 cells (p-value > 0.05).

### Analysis of SP phenotype

**Figure 4:**
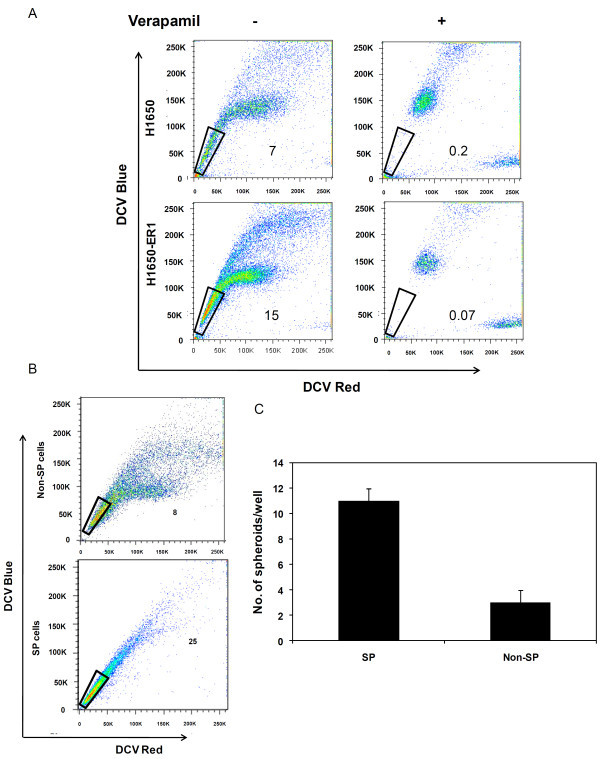
**Identification and analysis of H1650 and H1650-ER1 side population (SP) cells**. (A) The cells were stained with DCV dye blue-red profile. As a control, verapamil (50 μM) was added to the cells. SP cells were gated as the population of low DCV dye containing cells that disappeared upon verapamil treatment. A typical flow cytometry plot is shown where the SP fraction corresponds to 7% H1650 and 15% H1650-ER1 cells. Three independent experiments were carried out (B) H1650-ER1 SP and non SP cells were sorted and cultured for 10 days, stained with DCV dye and reanalyzed. SP cells generated SP and non SP fractions identical to H1650-ER1 cells while non SP cells generated mainly non SP cells. A representative plot is shown here. (C) Generation of tumor spheroids by SP and non SP cells. 6000 cells were seeded per well in 6 well plates and tumor spheroid generation was assessed by counting after 15 days. The error bars represent S.D. (n = 6).

Side population (SP) cells refer to cells which are highly enriched in stem cell activity. These cells are identified and/or isolated on the basis of their ability to efflux Hoechst 33342 or DyeCycle Violet (DCV) dye due to overexpression of ABCG2, an ATP binding cassette transporter [30]. We evaluated the existence of SPs in H1650 and H1650-ER1 cells by staining them with DCV dye to generate a DCV blue-red profile. As a control, verapamil, an ABCG2 specific inhibitor, was added. The SP gate was defined as region corresponding to cells that exhibited low DCV dye content in the absence of verapamil. Analysis of SPs in the parental H1650 cell line and the erlotinib resistant H1650-ER1 subline revealed differential SP fractions, ranging from 0.2 ± 0.01 and 4 ± 2 (with and without verapamil) for H1650 to 0.07 ± 0.05 and 15 ± 2.5 for H1650-ER1 cells (Figure 4A), suggesting EGFR-TKI exposure selectively enriched cells with stem cell activity (p-value < 0.05).

To investigate differentiation capability, FACS-sorted SP and non SP cells from H1650-ER1 were cultured under the same culture conditions for 10 days, restained with DCV dye and reanalyzed. Analysis indicates that sorted SP cells generated 20% SP cells upon subculture, demonstrating that SP cells can differentiate to non SP cells. Sorted non SP cells generated only 6% SP cells, which may have generated from the residual SP cells or transition of non-SP cells to SP cells (Figure 4B). We next evaluated the self renewal of SP and non SP cells by their spheroid formation ability. As shown in Figure 4C, SP cells gave rise to significantly higher number of spheroids as compared to non SP cells. These observations reveal the ability of SP cells to undergo asymmetrical division to self renew as well as generate differentiated tumor cells.

### Evaluation of in vitro tumorigenicity

**Figure 5:**
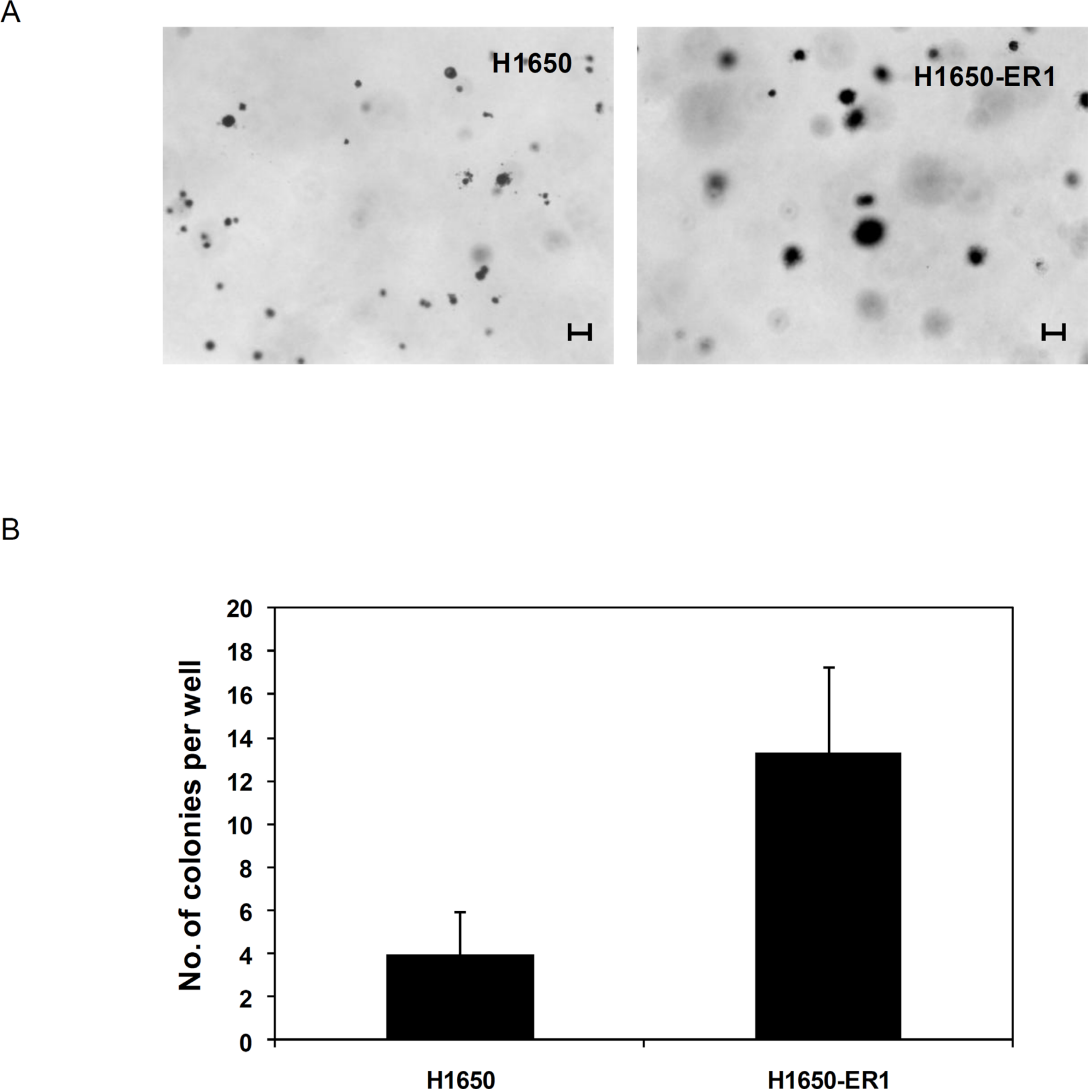
**Tumorigenicity of H1650 and H1650-ER1 cells was examined *in vitro *by a soft agar assay**. Dissociated cells resuspended in 0.35% agar were plated on 5% agar and the number of colonies counted after 15 days. (A) Images showing the colonies formed by H1650 and H1650-ER1 cells. The scale bar corresponds to 20 μm. (B) Quantification of colonies formed from 2500 cells in each well. The resistant subline formed a significantly higher number of colonies (p-value < 0.05). The error bars represent s.e.m. (n = 3).

A definite hallmark of CSCs is their tumorigenic potential. The ability of transformed cells to form colonies in soft agar is closely related to in vivo carcinogenesis and is often used as a surrogate *in vitro *assay for tumorigenicity [43,44]. To quantify colony forming efficiency, 2500 cells in 0.35% agar were seeded on top of 0.5% agar. The number of colonies greater than 20 μm was counted after 2 weeks. As illustrated in Figure 5A and 5B, H1650-ER1 cells formed a significantly higher number of colonies compared to H1650 cells, suggesting the resistant subline is comprised of higher number of cancer stem cell-like cells than the parental cells.

### Investigating the role of cancer cells with stem cell phenotypes in TKI resistance

**Figure 6:**
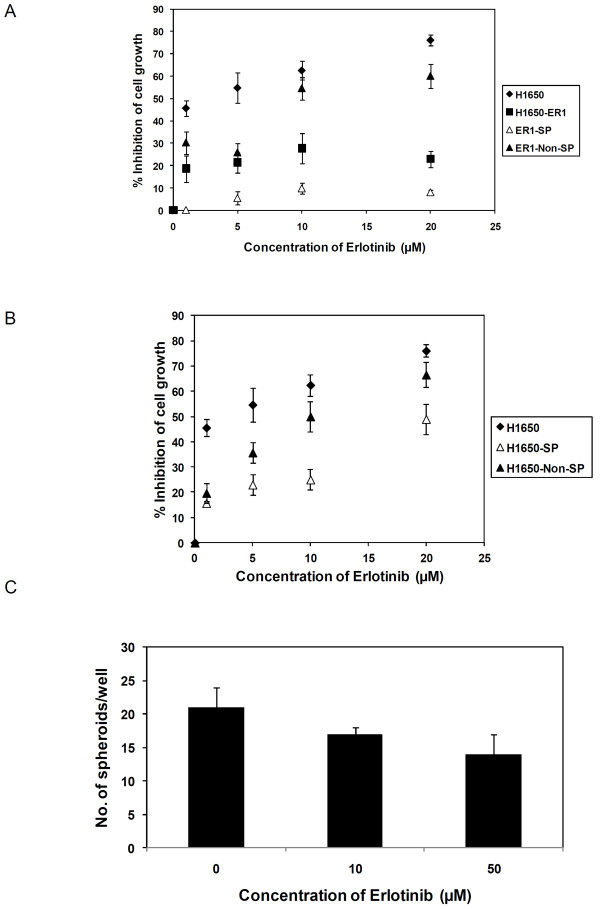
**Effect of erlotinib on cell growth**. (A) Dose-response curves of parental H1650, resistant subline H1650-ER1, sorted ER1-SP and ER1-non SP cells following incubation with varying concentrations of erlotinib for 48 hr. Each data point represents the mean of three independent experiments. The error bars represent s.e.m. (n = 3). (B) Dose-response curves of parental H1650, sorted H1650-SP and H1650 - non SP cells following incubation with varying concentrations of erlotinib for 48 hr. Each data point represents the mean of six replicates. The error bars represent S.D. (n = 6). (C) Quantification of spheroid formation by 6000 H1650-ER1 cells per well under the continuous presence of erlotinib. Each data point represents the mean of two independent experiments. The error bars represent s.e.m. (n = 3).

Our studies revealed that H1650-ER1 cells are enriched with cancer stem cell like cells. Next we investigated the role of these cells in inducing resistance to erlotinib therapy. Towards this aim, we determined whether SP cells preferentially survive erlotinib exposure as compared to non-SP cells. As shown in Figure 6A, higher viability at all erlotinib concentrations was observed in SP cells. Erlotinib inhibition of proliferation of non-SP cells matches that of H1650 parental cells closely. Next, we characterized the resistance phenotype of SP cells of H1650 parental cell line. As illustrated in Figure 6B, SP cells exhibited greater resistance to erlotinib insult than non SP cells, and similar resistance as the H1650-ER1 subline.

The resistance phenotype of these stem like cells was further confirmed by investigating spheroid forming ability of H1650-ER1 cells under continuous exposure to 10 μM and 50 μM of erlotinib (Figure 6C). The presence of erlotinib did not have a striking effect on spheroid formation frequency (17 ± 3 spheroids/6 × 10^3 ^cells without erlotinib, 13 ± 1 spheroids/6 × 10^3 ^cells with 10 μM erlotinib, 10 ± 3 spheroids/6 × 10^3 ^cells with 50 μM erlotinib, p-value > 0.05). These observations indicate that these putative cancer stem cells are inherently resistant to erlotinib treatment.

Similar to an earlier study which demonstrated the existence of an erlotinib resistant mesenchymal subpopulation expressing CD44^high^/CD24^low ^markers in different erlotinib naïve NSCLC cell lines and tumors [15], our study indicates that the lung cancer cell line H1650 consists of a population of putative cancer stem cells which are inherently resistant to erlotinib. Prolonged exposure of H1650 cells to erlotinib resulted in the selection of these cancer stem like cells in the erlotinib resistant H1650-ER1 cells, which in turn resulted in the acquisition of resistance to erlotinib.

### Detection of cancer stem-cell like cells in erlotinib resistant head and neck cancer sublines

**Figure 7:**
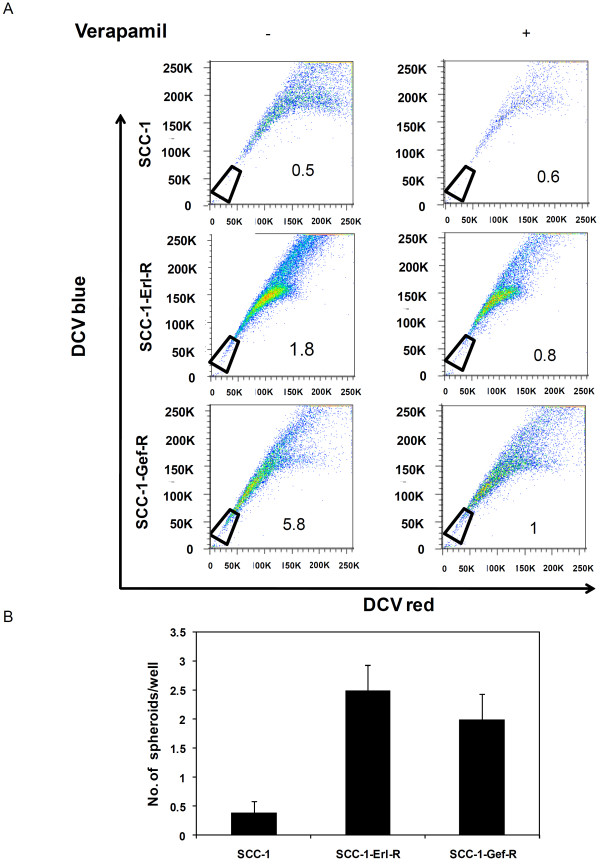
**Side population and tumor spheroid analysis of SCC-1, SCC-1-Erl-R and SCC-1-Gef-R cells**. (A) Side population (SP) analysis of SCC-1, SCC-1-Erl-R and SCC-1-Gef-R cells. (B) Quantification of spheroids generated per well. 6000 cells were seeded in each well of 6 well plates and spheroid generation was quantified after 15 days. Resistant sublines generated a significantly higher number of spheroids (p-value < 0.05). Each data point represents the mean of two independent experiments. The error bars represent s.e.m. (n = 3).

To exclude the possibility of occurrence of erlotinib resistance in generating cell populations with cancer stem cell properties only in H1650 cells, we investigated CSC properties in human head and neck squamous carcinoma cell line SCC-1 and EGFR TKI refractory sublines (SCC-1-Erl-R and SCC-1-Gef-R) [45]. Side population analysis revealed that the SCC-1 cell SP consisted of approximately 0.6% and 0.5% of cells in the presence and absence of verapamil, respectively, indicating that these cells did not contain a significant side population of stem cell like cells (Figure 7A). However, the SCC-1-Erl-R SP fraction contained 0.8% and 1.8% of cells and the SCC-1-Gef-R SP contained 1.0% and 5.8% of cells in the presence and absence of verapamil, highlighting the presence of drug-effluxing side population cells within the resistant sublines. Next, the ability of these cells to self-renew in spheroid culture was tested. As demonstrated in Figure 7B, when cultured under serum free non adherent conditions, a significantly increased number of spheroids was formed by the resistant sublines.

Collectively, our study indicates the presence of a population of cells with CSC traits in EGFR TKI naïve cancer cells, which are resistant to TKI therapy. So, while TKIs can inhibit kinase activity in differentiated cancer cells, they have little effect on putative CSCs. Prolonged exposure to these TKIs results in selection of cells with CSC phenotypes leading to acquisition of resistance towards EGFR TKI therapy.

## Conclusion

Our studies indicate that prolonged exposure of the NSCLC cell line H1650 to erlotinib selects for a subpopulation of erlotinib resistant cells which are enriched in stem cell markers and possess stem cell properties *in vitro*. A resistant subline, H1650-ER1, expressed enhanced level of stem cell surface markers and also exhibited increased mRNA expression of transcription factors OCT3/4, NANOG, SOX-2, and ID2. H1650-ER1 cells also showed increased self renewal and the ability to differentiate, considered fundamental properties of CSCs. Our studies indicated that continuous exposure of H1650 cells to erlotinib selected for cells with CSC traits. Furthermore, these cells were found to be less sensitive to erlotinib treatment as determined by cell viability and tumor spheroid formation in the presence of different concentrations of erlotinib. To ascertain that the existence of CSC like cells in H1650 and corresponding enrichment upon erlotinib treatment in H1650-ER1 cells is not specific to H1650 cell line, presence of cells with CSC traits was also investigated in human head and neck squamous carcinoma cell line SCC-1 and EGFR TKI refractory sublines (SCC-1-Erl-R and SCC-1-Gef-R). We also demonstrated the existence of putative CSCs in SCC-1 as well as SCC-1-Erl-R and SCC-1-Gef-R cells via side population analysis and tumor spheroid formation assay.

In conclusion, our study provides compelling evidence that resistance to molecular targeted therapies may be due to cancer stem cell-like cells which are intrinsically resistant to erlotinib treatment. These cells are present even before erlotinib treatment. However, erlotinib treatment selects for these cells and enrichment of cells with CSC markers and *in vitro *phenotypes results in the acquisition of resistance. The study suggests that supplementation of EGFR kinase inhibition with strategies to target cancer stem cell-like populations may increase effectiveness of EGFR inhibition therapies.

## Competing interests

The authors declare that they have no competing interests.

## Authors' contributions

GG designed and performed the experiments and also wrote the manuscript. XL assisted RT-PCR, immunofluorescence, and flow cytometry experiments. SK and SP designed experiments and reviewed the manuscript. All authors read and approved the final version.

## Pre-publication history

The pre-publication history for this paper can be accessed here:

http://www.biomedcentral.com/1471-2407/12/95/prepub

## References

[B1] Sebolt-Leopold JS English JM Mechanisms of drug inhibition of signalling molecules Nature 2006 441 457 62 10.1038/nature04874 16724058

[B2] Laskin JJ Sandler AB Epidermal growth factor receptor: a promising target in solid tumours Cancer Treat Rev 2004 30 1 17 10.1016/j.ctrv.2003.10.002 14766123

[B3] Madhusudan S Ganesan TS Tyrosine kinase inhibitors in cancer therapy Clin Biochem 2004 37 618 635 10.1016/j.clinbiochem.2004.05.006 15234243

[B4] Yarden Y The EGFR family and its ligands in human cancer: signaling mechanisms and therapeutic opportunities Eur J Cancer 2001 37 S3 S8 11597398 10.1016/s0959-8049(01)00230-1

[B5] Vlahovic G Crawford J Activation of tyrosine kinases in cancer The Oncologist 2003 8 531 538 10.1634/theoncologist.8-6-531 14657531

[B6] Inoue A Suzuki T Fukuhara T Maemondo M Kimura Y Morikawa N Prospective phase II study of gefitinib for chemotherapy-naïve patients with advanced non-small-cell lung cancer with epidermal growth factor receptor gene mutations J Clin Oncol 2006 24 3340 3346 10.1200/JCO.2005.05.4692 16785471

[B7] Shepherd FA Pereira JR Ciuleanu T Tan EH Hirsh V Thongprasert S Erlotinib in previously treated non-small-cell lung cancer N Engl J Med 2005 353 123 132 10.1056/NEJMoa050753 16014882

[B8] Sequist LV Bell DW Lynch TJ Haber DA Molecular predictors of response to epidermal growth factor receptor antagonists in non-small-cell lung cancer J Clin Oncol 2007 25 587 595 10.1200/JCO.2006.07.3585 17290067

[B9] Rossell R Moran T Queralt C Porta R Cardenal F Majem M Screening for Epidermal Growth Factor Receptor mutations in lung Cancer N Engl J Med 2009 361 958 967 10.1056/NEJMoa0904554 19692684

[B10] Kobayashi S Boggon TJ Dayaram T Janne PA Kocher O Meyerson M EGFR mutation and resistance of non-small-cell lung cancer to gefitinib N Engl J Med 2005 352 786 792 10.1056/NEJMoa044238 15728811

[B11] Pao W Miller VA Politi KA Riely GJ Somwar R Zakowski MF Acquired resistance of lung adenocarcinomas to gefitinib or erlotinib is associated with a second mutation in the EGFR kinase domain PLoS Med 2005 2 e73 10.1371/journal.pmed.0020073 15737014 PMC549606

[B12] Bean J Brennan C Shih JY Riely G Viale A Wang L MET amplification occurs with or without T790M mutations in EGFR mutant lung tumors with acquired resistance to gefitinib or erlotinib Proc Natl Acad Sci USA 2007 104 20932 20937 10.1073/pnas.0710370104 18093943 PMC2409244

[B13] Engelman JA Zejnullahu K Mitsudomi T Song Y Hyland C Park JO MET amplification leads to gefitinib resistance in lung cancer by activating ERBB3 signaling Science 2007 316 1039 1043 10.1126/science.1141478 17463250

[B14] Kwak EL Sordella R Bell DW Godin-Heymann N Okimoto RA Brannigan BW Irreversible inhibitors of the EGF receptor may circumvent acquired resistance to gefitinib Proc Natl Acad Sci USA 2005 102 7665 7670 10.1073/pnas.0502860102 15897464 PMC1129023

[B15] Yao Z Fenoglio S Gao DC Camiolo M Stiles B Lindsted T TGF-beta IL-6 axis mediates selective and adaptive mechanisms of resistance to molecular targeted therapy in lung cancer Proc Natl Acad Sci USA 2010 107 15535 15540 10.1073/pnas.1009472107 20713723 PMC2932568

[B16] Thomson S Buck E Petti F Griffin G Brown E Ramnarine N Epithelial to mesenchymal transition is a determinant of sensitivity of non-small-cell lung carcinoma cell lines and xenografts to epidermal growth factor receptor inhibition Cancer Res 2005 65 9455 9462 10.1158/0008-5472.CAN-05-1058 16230409

[B17] Thomson S Petti F Sujka-Kwok I Epstein D Haley JD Kinase switching in mesenchymal-like non-small cell lung cancer lines contributes to EGFR inhibitor resistance through pathway redundancy Clin Exp Metastasis 2008 25 843 854 10.1007/s10585-008-9200-4 18696232

[B18] Uramoto H Iwata T Onitsuka T Shimokawa H Hanagiri T Oyama T Epiethelial-mesenchymal transition in EGFR-TKI acquired resistant lung adenocarcinoma Anticancer Res 2010 30 2513 2518 20682976

[B19] Mani SA Guo W Liao MJ Eaton EN Ayyanan A Zhou AY The epithelial-mesenchymal transition generates cells with properties of stem cells Cell 2008 133 704 715 10.1016/j.cell.2008.03.027 18485877 PMC2728032

[B20] Bonnet D Dick JE Human acute myeloid leukemia is organized as a hierarchy that originates from a primitive hematopoietic cell Nat Med 1997 3 730 737 10.1038/nm0797-730 9212098

[B21] Al-Hajj M Wicha MS Benito-Hernandez A Morrison SJ Clarke MF Prospective identification of tumorigenic breast cancer cells Proc Natl Acad Sci USA 2003 100 3983 3988 10.1073/pnas.0530291100 12629218 PMC153034

[B22] Singh SK Hawkins C Clarke ID Squire JA Bayani J Hide T Identification of human brain tumour initiating cells Nature 2004 432 396 401 10.1038/nature03128 15549107

[B23] Bertolini G Roz L Perego P Tortoreto M Fontanella E Gatti L Highly tumorigenic lung cancer CD133+ cells display stem-like features and are spared by cisplatin treatment Proc Natl Acad Sci USA 2009 106 16281 16286 10.1073/pnas.0905653106 19805294 PMC2741477

[B24] Eramo A Haas TL De Maria R Lung cancer stem cells: tools and targets to fight lung cancer Oncogene 2010 29 4625 4635 10.1038/onc.2010.207 20531299

[B25] Gu G Yuan J Wills M Kasper S Prostate cancer cells with stem cell characteristics reconstitute the original human tumor in vivo Cancer Res 2007 67 4807 4815 10.1158/0008-5472.CAN-06-4608 17510410

[B26] Li C Heidt DG Dalerba P Burant CF Zhang L Adsay V Identification of pancreatic cancer stem cells Cancer Res 2007 67 1030 1037 10.1158/0008-5472.CAN-06-2030 17283135

[B27] Sullivan JP Minna JD Shay JW Evidence for self-renewing lung cancer stem cells and their implications in tumor initiation, progression, and targeted therapy Cancer Metastasis Rev 2010 29 61 72 10.1007/s10555-010-9216-5 20094757 PMC2864581

[B28] Wicha MS Liu S Dontu G Cancer stem cells: an old idea--a paradigm shift Cancer Res 2006 66 1883 1890 discussion 95-6 10.1158/0008-5472.CAN-05-3153 16488983

[B29] Goodell MA Brose K Paradis G Conner AS Mulligan RC Isolation and functional properties of murine hematopoietic stem cells that are replicating in vivo J Exp Med 1996 183 1797 1806 10.1084/jem.183.4.1797 8666936 PMC2192511

[B30] Telford WG Bradford J Godfrey W Robey RW Bates SE Side population analysis using a violet-excited cell-permeable DNA binding dye Stem Cells 2007 25 1029 1036 10.1634/stemcells.2006-0567 17185610

[B31] Ghosh G Yan X Lee AG Kron SJ Palecek SP Quantifying the sensitivities of EGF receptor (EGFR) tyrosine kinase inhibitors in drug resistant non-small cell lung cancer (NSCLC) cells using hydrogel-based peptide array Biosens Bioelectron 2010 26 424 431 10.1016/j.bios.2010.07.106 20729058 PMC2946430

[B32] Yauch RL Januario T Eberhard DA Cavet G Zhu W Fu L Epithelial versus mesenchymal phenotype determines *in vitro *sensitivity and predicts clinical activity of erlotinib in lung cancer patients Clin Cancer Res 2005 11 8686 8698 10.1158/1078-0432.CCR-05-1492 16361555

[B33] Rho JK Choi YJ Lee JK Ryoo BY Na II Yang SH Epithelial to mesenchymal transition derived from repeated exposure to gefitinib determines the sensitivity to EGFR inhibitors in A549, a non-small cell lung cancer cell line Lung Cancer 2009 63 219 226 10.1016/j.lungcan.2008.05.017 18599154

[B34] Beier D Hau P Proescholdt M Lohmeier A Wischhusen J Oefner PJ CD133(+) and CD133(-) glioblastoma-derived cancer stem cells show differential growth characteristics and molecular profiles Cancer Res 2007 67 4010 4015 10.1158/0008-5472.CAN-06-4180 17483311

[B35] Eramo A Lotti F Sette G Pillozi E Biffoni M Virgillo AD Identification and expansion of the tumorigenic lung cancer stem cell population Cell Death and Differentiation 2008 15 504 514 10.1038/sj.cdd.4402283 18049477

[B36] Hermann PC Huber SL Herrler T Aicher A Ellwart JW Guba M Distinct populations of cancer stem cells determine tumor growth and metastatic activity in human pancreatic cancer Cell Stem Cell 2007 1 313 323 10.1016/j.stem.2007.06.002 18371365

[B37] Miki J Furusato B Li H Gu Y Takahashi H Egawa S Identification of putative stem cell markers, CD133 and CXCR4, in hTERT-immortalized primary nonmalignant and malignant tumor-derived human prostate epithelial cell lines and in prostate cancer specimens Cancer Res 2007 67 3153 3161 10.1158/0008-5472.CAN-06-4429 17409422

[B38] Lengner CJ Welstead GG Jaenisch R The pluripotency of regulator OCT4: A role in somatic cells? Cell Cycle 2008 7 725 728 10.4161/cc.7.6.5573 18239456

[B39] Chiou SH Wang ML Chou YT Chen CJ Hong CF Hseih WJ Coexpression of OCT4 and Nanog enhances malignancy in lung adenocarcinoma by inducing cancer stem cell like properties and epithelial mesenchymal transdifferentiation Cancer Res 2010 70 10433 10444 10.1158/0008-5472.CAN-10-2638 21159654

[B40] Park IK Qian D Kiel M Becker MW Pihalja M Weissman IL Bmi-1 is required for maintenance of adult self-renewing haematopoietic stem cells Nature 2003 423 302 305 10.1038/nature01587 12714971

[B41] Niwa H Burdon T Chambers I Smith A Self-renewal of pluripotent embryonic stem cells is mediated via activation of STAT3 Genes Dev 1998 12 2048 2060 10.1101/gad.12.13.2048 9649508 PMC316954

[B42] Chambers I Colby D Robertson M Nichols J Lee S Tweedie S Functional expression cloning of Nanog, a pluripotency sustaining factor in embryonic stem cells Cell 2003 113 643 655 10.1016/S0092-8674(03)00392-1 12787505

[B43] Colburn NH Bruegge WF Bates JR Gray RH Rossen JD Kelsey WH Correlation of anchorage-independent growth with tumorigenicity of chemically transformed mouse epidermal cells Cancer Res 1978 38 624 634 626967

[B44] Hwang-Verslues WW Kuo WH Chang PH Pan CC Wang HH Tsai ST Multiple lineages of human breast cancer stem/progenitor cells identified by profiling with stem cell markers PLoS One 2009 4 e8377 10.1371/journal.pone.0008377 20027313 PMC2793431

[B45] Benavente S Huang S Armstrong EA Chi A Hsu KT Wheeler DL Establishment and characterization of a model of acquired resistance to epidermal growth factor receptor targeting agents in human cancer cells Clin Cancer Res 2009 15 1585 1592 10.1158/1078-0432.CCR-08-2068 19190133 PMC2903727

